# Crystal structure and Hirshfeld surface analysis of methyl 4′-amino-3′,5′-di­cyano-2,2′′-dioxodi­spiro[indoline-3,1′-cyclo­pentane-2′,3′′-indolin]-3′-ene-5′-carboximidate with an unknown solvent

**DOI:** 10.1107/S2056989022005370

**Published:** 2022-05-24

**Authors:** Farid N. Naghiyev, Victor N. Khrustalev, Elena A. Fortalnova, Mehmet Akkurt, Ali N. Khalilov, Ajaya Bhattarai, İbrahim G. Mamedov

**Affiliations:** a Department of Chemistry, Baku State University, Z. Khalilov str. 23, Az, 1148 Baku, Azerbaijan; b Peoples’ Friendship University of Russia (RUDN University), Miklukho-Maklay St. 6, Moscow, 117198, Russian Federation; c N. D. Zelinsky Institute of Organic Chemistry RAS, Leninsky Prosp. 47, Moscow, 119991, Russian Federation; d Department of Physics, Faculty of Sciences, Erciyes University, 38039 Kayseri, Turkey; e "Composite Materials" Scientific Research Center, Azerbaijan State Economic University (UNEC), H. Aliyev str. 135, Az 1063, Baku, Azerbaijan; f Department of Chemistry, M.M.A.M.C (Tribhuvan University) Biratnagar, Nepal; Indian Institute of Science Education and Research Bhopal, India

**Keywords:** crystal structure, di­spiro­[cyclo­pent-3-ene]bis­oxindoles, hydrogen bond, Hirshfeld surface analysis, mol­ecular conformation

## Abstract

In the title compound, C_23_H_16_N_6_O_3_, the central five-membered cyclo­pentene ring adopts an envelope conformation while the five-membered spiro 2,3-di­hydro-1*H*-pyrrole rings exhibit twisted envelope and envelope conformations. One of the 1,3-di­hydro-2*H*-indol-2-one units is in an axial position and the other is in a bis­ectional position. The methyl methanimidate unit is in an equatorial position. The crystal structure of the title compound is consolidated by inter­molecular N—H⋯N, N—H⋯O and C—H⋯O hydrogen bonds, forming a three dimensional network.

## Abstract 2

One of the 1,3-di­hydro-2*H*-indol-2-one units is in an axial position, while the other is in a bis­ectional position relative to the central five-membered cyclo­pentene ring. The methyl methanimidate unit is in an equatorial position. The crystal structure is consolidated by inter­molecular N—H⋯N, N—H⋯O and C—H⋯O hydrogen bonding, forming a three-dimensional network.

## 1. Chemical context

**Figure 1:**
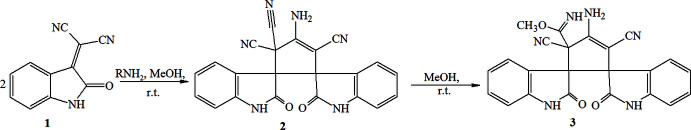
The formation of **3**.

Functionalized carbo- and heterocycles are of great inter­est in the fields of organic synthesis, catalysis, material science and medicinal chemistry (Zubkov *et al.*, 2018 ▸; Shikhaliyev *et al.*, 2019 ▸; Viswanathan *et al.*, 2019 ▸; Gurbanov *et al.*, 2020 ▸). Cyclization of carbo- and heterocycles with the participation of malono­nitrile to obtain spiro compounds has been reported in the literature. (Zhu *et al.*, 2016 ▸; Tan *et al.*, 2020 ▸). In addition, it is known that the reaction of Hantzsch ester with two mol­ecules of 2-(2-oxoindolin-3-yl­idene)malono­nitrile, **1**, leads to the formation of di­spiro­[cyclo­pent-3-ene]bis­oxindoles, **2** (Shanthi & Perumal, 2008 ▸). We found that one of the nitrile groups of di­spiro­[cyclo­pent-3-ene]bis­oxindole tricarbo­nitrile **2** reacted with the methanol (solvent) gave rise to compound **3** (Fig. 1 ▸).

Thus, in the framework of ongoing structural studies (Safavora *et al.*, 2019 ▸; Aliyeva *et al.*, 2011 ▸; Mamedov *et al.*, 2022 ▸), we report the crystal structure and Hirshfeld surface analysis of the title compound, methyl 4′-amino-3′,5′-di­cyano-2,2′′-dioxodi­spiro­[indoline-3,1′-cyclo­pentane-2′,3′′-indolin]-3′-ene-5′-carbimidate, which has an unknown solvent. 
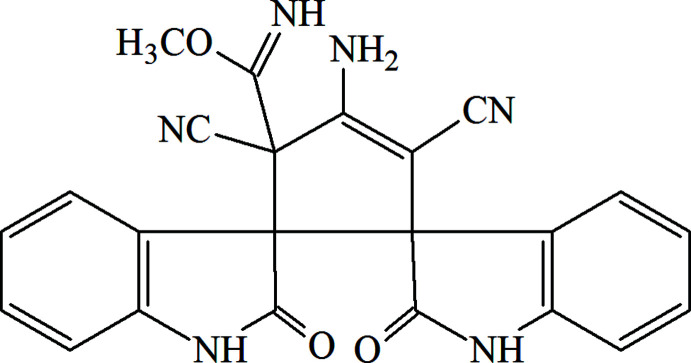


## 2. Structural commentary

**Figure 2:**
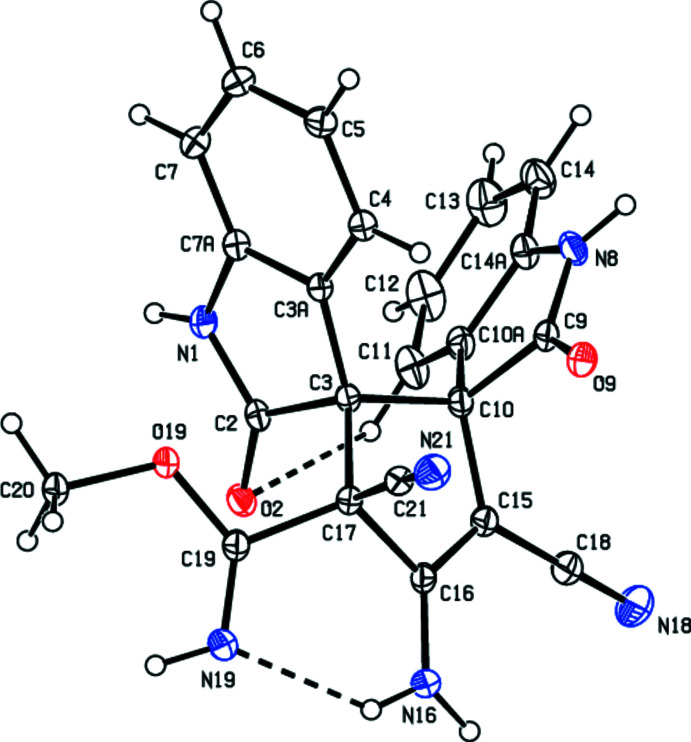
The mol­ecular structure of the title compound. Displacement ellipsoids are drawn at the 30% probability level.

The title compound (Fig.2) crystallizes in the monoclinic space group *P*2_1_/*c* with *Z* = 4. The N1/O2/C2/C3/C3*A*/C4–C7/C7*A* 1,3-di­hydro-2*H*-indol-2-one unit, which is attached to C3, makes a dihedral angle of 1.71 (6)° with the mean plane of the central five-membered cyclo­pentene ring (C3/C10/C15–C17). The N8/C9/C10/C10*A*/C14*A* 1,3-di­hydro-2*H*-indol-2-one unit, which is attached to C10, forms a dihedral angle of 57.50 (4)° with the other 1,3-di­hydro-2*H*-indol-2-one unit. The methyl methanimidate unit, which is attached to C17, is in an equatorial position. The conformation of the title mol­ecule, (Fig. 2 ▸), is fixed because of the weak intra­molecular N16—H16*A*⋯N19 [2.079 (19) Å, 132.5 (16)°] and C11—H11⋯O2 [2.53 Å, 123°] hydrogen bonds, which close the six- and seven-membered rings with graph-set notations *S*(6) and *S*(7), respectively (Bernstein *et al.*, 1995 ▸; Table 1 ▸).

The central five-membered cyclo­pentene ring (C3/C10/C15–C17) adopts an envelope conformation with the flap atom, C3, lying 0.181 (1) Å out of the plane defined by the remaining atoms. The puckering parameters (Cremer & Pople, 1975 ▸) are *Q*(2) = 0.2915 (14) Å, φ(2) = 175.0 (3)°. The five-membered spiro 2,3-di­hydro-1*H*-pyrrole rings (N1/C2/C3/C3*A*/C7*A* and N8/C9/C10/C10*A*/C14*A*) exhibit a twisted envelope conformation on bond C2–C3 and an envelope conformation with atom C10 as a flap, respectively. Their puckering parameters are *Q*(2) = 0.0864 (13) Å, φ(2) = 62.5 (9)° and *Q*(2) = 0.0810 (14) Å, φ(2) = 64.7 (10)°, respectively.

## 3. Supra­molecular features and Hirshfeld surface analysis

**Figure 3:**
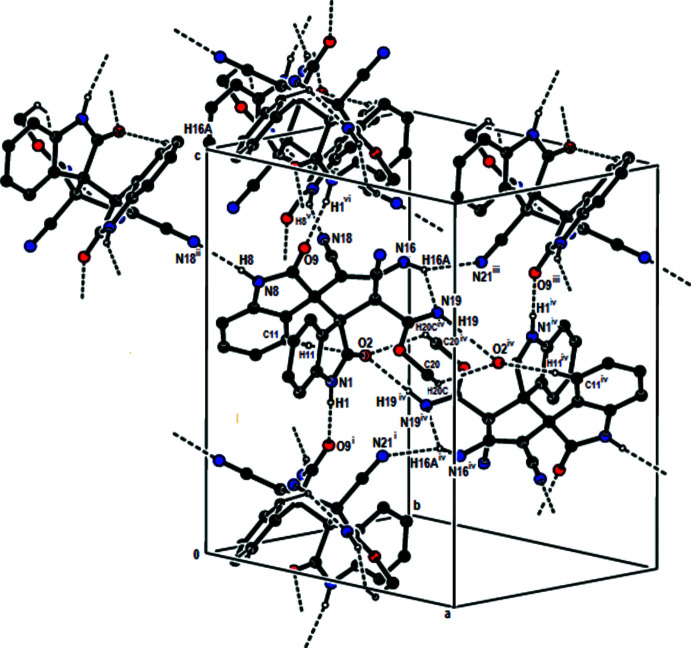
A general view of the packing and N—H⋯N, N—H⋯O and C—H⋯O hydrogen bonding of the title compound in the unit cell. The hydrogen atoms not involved in the hydrogen bonds have been omitted for clarity. Symmetry codes: (i) *x*, −*y* + 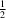 , *z* − 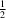 ; (ii) −*x*, *y* − 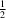 , −*z* + 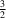 ; (iii) −*x* + 1, *y* + 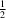 , −*z* + 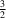 ; (iv) −*x* + 1, −*y* + 1, −*z* + 1; (v) −*x*, 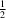 + *y*, 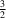 − *z*; (vi) *x*, 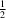 − *y*, 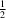 + *z*.

In the crystal, pairs of mol­ecules are linked by inter­molecular N19—-H19⋯O2(−*x* + 1, −*y* + 1, −*z* + 1) hydrogen bonds into inversion dimers with an 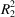 (14) ring motif (Bernstein *et al.*, 1995 ▸). Weak inter­molecular C20—-H20*C*⋯O2(−*x* + 1, −*y* + 1, −*z* + 1) and intra­molecular N16—H16*A*⋯N19 (*x*, *y*, *z*) hydrogen bonds also form an *S*(6) 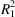 (6) 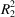 (14) 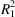 (6)*S*(6) ring motif system between these dimer mol­ecules. Futhermore, these dimers are linked by N8—H8⋯N18(−*x*, *y* − 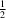 , −*z* + 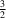 ) hydrogen bonds in the directions of both base diagonals of the *ab* plane of the unit cell, forming sheets parallel to the (001) plane. These layers are also connected along the *c*-axis direction by N1—H1⋯O9 (*x*, −*y* + 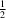 , *z* − 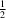 ) and N16—H16*A*⋯N21 (−*x* + 1, *y* + 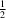 , −*z* + 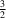 ) hydrogen bonds (Table 1 ▸, Fig. 3 ▸). The three-dimensional hydrogen-bonded network thus formed keeps the crystal structure stable.

**Figure 4:**
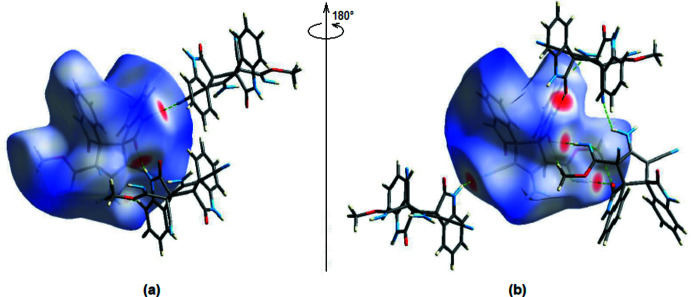
The three-dimensional Hirshfeld surface of the title compound plotted over *d*_norm_ in the range −0.6120 to 2.8879 a.u. The N—H⋯N and N—H⋯O hydrogen bonds are shown.

Hirshfeld surface analysis can be used to qualitatively visualize the main inter­actions between mol­ecules (Spackman & Jayatilaka, 2009 ▸). *CrystalExplorer17.5* (Turner *et al.*, 2017 ▸) was used to map the normalized contact distance (*d*_norm_). On the Hirshfeld surfaces, the most notable inter­actions (short contact areas) are represented in red, whereas long contacts are displayed in blue. Fig. 4 ▸ depicts the three-dimensional Hirshfeld surface overlaid over *d*_norm_ in the range −0.6120 (red) to +2.8879 (blue) a.u.

**Figure 5:**
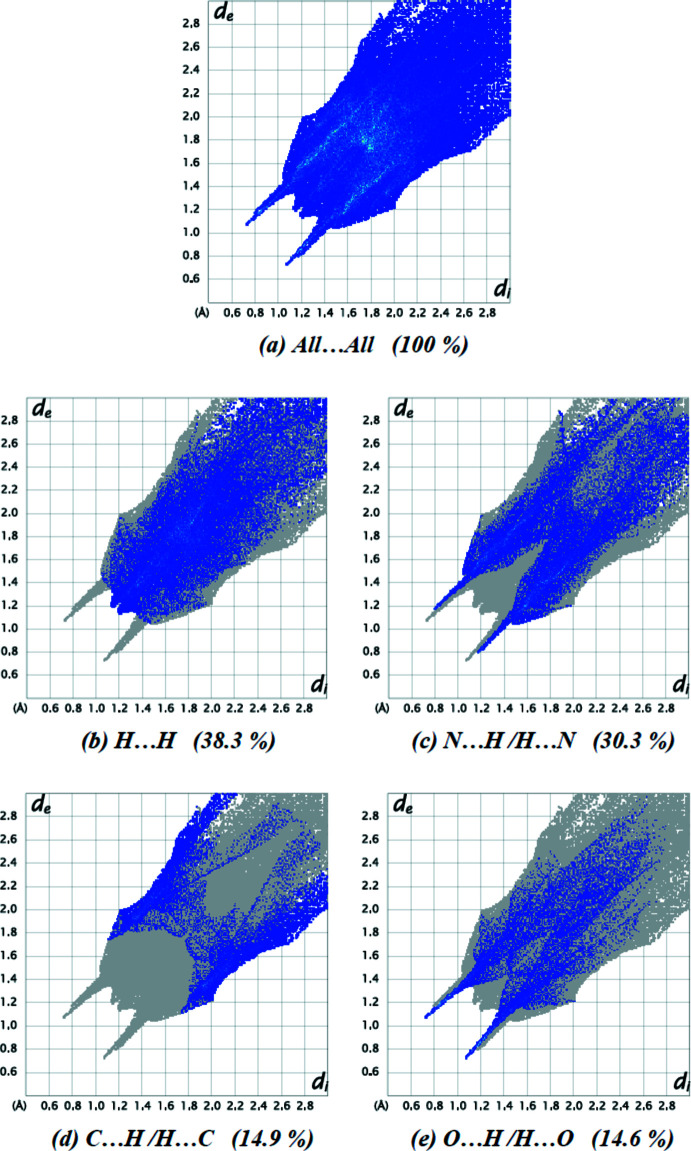
The two-dimensional fingerprint plots for the title compound, showing (*a*) all inter­actions, and delineated into (*b*) H⋯H, (*c*) N⋯H/H⋯N, (*d*) C⋯H/H⋯C and (*e*) O⋯H/H⋯O inter­actions [*d*_e_ and *d*_i_ represent the distances from a point on the Hirshfeld surface to the nearest atoms outside (external) and inside (inter­nal) the surface, respectively].

Fingerprint plots were created to indicate inter­molecular surface bond distances, with regions highlighted for N⋯H/H⋯N and O⋯H/H⋯O inter­actions (Table 1 ▸, Fig. 5 ▸). Such connections contribute 30.3% and 14.6%, respectively, of the surface area. The very low number of C⋯H/H⋯C connections (14.9%) shows that these inter­actions play a minor role in crystal-packing consolidation. The contribution to the surface area for H⋯H contacts is 38.3%. Other weak contacts contribute only 1.0% (C⋯C), 0.5% (N⋯C/C⋯N), 0.2% (O⋯O), 0.1% (N⋯N) and 0.1% (O⋯C/C⋯O) to the Hirshfeld surface.

## 4. Database survey

The compound most closely related to the 2,8-di­aza­dispiro[4.0.4^6^.3^5^]trideca-3,9,11-triene unit of the title compound was found to be 4′-amino-2,2′′-dioxo-1,1′′,2,2′′-tetra­hydro-3′*H*-di­spiro­[indole-3,1′-cyclo­pent[4]ene-2′,3′′-indole]-3′,3′-dicarbo­nitrile dihydrate (GITGUM; Gayathri *et al.*, 2008 ▸), which crystallizes in the ortho­rhom­bic space group, *P*na2_1_. The cyclo­pentene ring adopts an envelope conformation, with the spiro C atom bonded to the di­cyano-substituted C atom deviating by 0.437 (2) Å from the plane of the remaining four atoms in the ring. The dihedral angle between the two indole groups is 60.1 (1)°. The structure contains inter­molecular N— H⋯O hydrogen bonds involving the indole groups and O—H⋯O and O—H⋯N hydrogen bonds involving the water mol­ecules.

## 5. Synthesis and crystallization

A solution of 2-(2-oxoindolin-3-yl­idene)malono­nitrile (0.99 g; 5.1 mmol) and furfuryl­amine (0.5 g; 5.2 mmol) in methanol (25 mL) was stirred for 10 minutes and was kept in room temperature for 96 h. Then 15 mL of methanol were removed from the reaction mixture, which was left overnight. The precipitated crystals were separated by filtration and recrystallized from ethanol/water (1:1) solution (yield 46%; m.p. 574–575 K).

^1^H NMR (300 MHz, DMSO-*d*_6_, p.p.m.): 3.78 (*s*, 3H, CH_3_); 6.62–7.26 (*m*, 8H, 8CH_arom._); 7.69 (*s*, 2H, NH_2_); 8.87 (*s*, 1H, NH); 10.56 (*s*, 1H, NH), 10.62 (*s*, 1H, NH). ^13^C NMR (75 MHz, DMSO-*d*_6_, ppm): 53.66 (OCH_3_), 54.56 (C_quat._), 56.63 (C_quat._), 75.06 (=C_quat_), 76.72 (=C_quat_), 109.96 (CH_arom._), 110.18 (CH_arom._), 116.32 (CN), 116.83 (CN), 122.08 (CH_arom._), 122.63 (CH_arom._), 124.17 (C_arom._), 124.41 (C_arom._), 126.07 (CH_arom._), 126.62 (CH_arom._), 130.27 (CH_arom._), 130.65 (CH_arom._), 143.14 (C_arom._), 143.31 (C_arom._), 159.57 (=C_quat._), 160.18 (=C_quat._), 175.07 (O=C—NH), 177.32 (O=C—NH).

## 6. Refinement

Crystal data, data collection and structure refinement details are summarized in Table 2 ▸. C-bound H atoms were positioned geometrically (C—H = 0.95–0.98 Å) and included as riding contributions with isotropic displacement parameters fixed at 1.2*U*_eq_(C) (1.5 for methyl groups). The N-bound H atoms were found in difference-Fourier maps and their coordinates refined with *U*_iso_(H)=1.2*U*_eq_(N). The residual electron density was difficult to model and therefore the SQUEEZE routine (Spek, 2015 ▸) in *PLATON* (Spek, 2020 ▸) was used to remove the contribution of the electron density in the solvent region from the intensity data and the solvent-free model was employed for the final refinement. The solvent formula mass and unit-cell characteristics were not taken into account during refinement. The cavity of volume *ca* 404.2 Å^3^ (*ca* 17.5% of the unit-cell volume) contains approximately 101 electrons. A suitable solvent with this electron number may be about four ethanol mol­ecules per unit cell.

## Supplementary Material

Crystal structure: contains datablock(s) I. DOI: 10.1107/S2056989022005370/dx2045sup1.cif

Structure factors: contains datablock(s) I. DOI: 10.1107/S2056989022005370/dx2045Isup2.hkl

CCDC reference: 2173900

Additional supporting information:  crystallographic information; 3D view; checkCIF report

## Figures and Tables

**Table 1:** Hydrogen-bond geometry (Å, °)

*D*—H⋯*A*	*D*—H	H⋯*A*	*D*⋯*A*	*D*—H⋯*A*
N1—H1⋯O9^i^	0.860 (19)	1.963 (18)	2.8145 (14)	170.0 (16)
N8—H8⋯N18^ii^	0.866 (19)	2.110 (18)	2.9270 (19)	157.1 (17)
N16—H16*A*⋯N19	0.883 (19)	2.079 (19)	2.7531 (17)	132.5 (16)
N16—H16*A*⋯N21^iii^	0.883 (19)	2.684 (19)	3.1854 (17)	117.1 (14)
N19—H19⋯O2^iv^	0.894 (19)	2.130 (19)	2.9912 (15)	161.6 (17)
C11—H11⋯O2	0.95	2.53	3.1499 (18)	123
C20—H20*C*⋯O2^iv^	0.98	2.56	3.1938 (16)	123

Symmetry codes: (i) 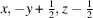 ; (ii) 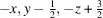 ; (iii) 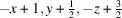 ; (iv) 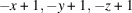 .

**Table 2:** Experimental details

Crystal data	
Chemical formula	C_23_H_16_N_6_O_3_
*M*_r_	424.42
Crystal system, space group	Monoclinic, *P*2_1_/*c*
Temperature (K)	100
*a*, *b*, *c* (Å)	12.0085 (1), 12.4719 (1), 15.4909 (1)
β (°)	94.489 (1)
*V* (Å^3^)	2312.94 (3)
*Z*	4
Radiation type	Cu *K*α
μ (mm^−1^)	0.70
Crystal size (mm)	0.15 × 0.12 × 0.10

Data collection	
Diffractometer	XtaLAB Synergy, Dualflex, HyPix
Absorption correction	Multi-scan (*CrysAlis PRO*; Rigaku OD, 2021 ▸)
*T*_min_, *T*_max_	0.891, 0.927
No. of measured, independent and observed [*I* > 2σ(*I*)] reflections	31502, 5011, 4771
*R*_int_	0.038
(sin θ/λ)_max_ (Å^−1^)	0.638

Refinement	
*R*[*F* ^2^ > 2σ(*F* ^2^)], *wR*(*F* ^2^), *S*	0.046, 0.128, 1.04
No. of reflections	5011
No. of parameters	305
H-atom treatment	H atoms treated by a mixture of independent and constrained refinement
Δρ_max_, Δρ_min_ (e Å^−3^)	0.35, −0.25

Computer programs: *CrysAlis PRO* (Rigaku OD, 2021 ▸), *SHELXT2014/5* (Sheldrick, 2015*a* ▸), *SHELXL2018/3* (Sheldrick, 2015*b* ▸), *ORTEP-3 for Windows* (Farrugia, 2012 ▸) and *PLATON* (Spek, 2020 ▸).

## supplementary crystallographic information

### Crystal data

**Table:**

C_23_H_16_N_6_O_3_	*F*(000) = 880
*M**_r_* = 424.42	*D*_x_ = 1.219 Mg m^−^^3^
Monoclinic, *P*2_1_/*c*	Cu *K*α radiation, λ = 1.54184 Å
*a* = 12.0085 (1) Å	Cell parameters from 22193 reflections
*b* = 12.4719 (1) Å	θ = 3.7–79.3°
*c* = 15.4909 (1) Å	µ = 0.70 mm^−^^1^
β = 94.489 (1)°	*T* = 100 K
*V* = 2312.94 (3) Å^3^	Prism, colourless
*Z* = 4	0.15 × 0.12 × 0.10 mm

### Data collection

**Table:**

XtaLAB Synergy, Dualflex, HyPix diffractometer	4771 reflections with *I* > 2σ(*I*)
Radiation source: micro-focus sealed X-ray tube	*R*_int_ = 0.038
φ and ω scans	θ_max_ = 79.7°, θ_min_ = 3.7°
Absorption correction: multi-scan (CrysAlisPro; Rigaku OD, 2021)	*h* = −15→14
*T*_min_ = 0.891, *T*_max_ = 0.927	*k* = −15→15
31502 measured reflections	*l* = −16→19
5011 independent reflections	

### Refinement

**Table:**

Refinement on *F*^2^	Primary atom site location: difference Fourier map
Least-squares matrix: full	Secondary atom site location: difference Fourier map
*R*[*F*^2^ > 2σ(*F*^2^)] = 0.046	Hydrogen site location: mixed
*wR*(*F*^2^) = 0.128	H atoms treated by a mixture of independent and constrained refinement
*S* = 1.04	*w* = 1/[σ^2^(*F*_o_^2^) + (0.0719*P*)^2^ + 1.0231*P*] where *P* = (*F*_o_^2^ + 2*F*_c_^2^)/3
5011 reflections	(Δ/σ)_max_ < 0.001
305 parameters	Δρ_max_ = 0.35 e Å^−^^3^
0 restraints	Δρ_min_ = −0.25 e Å^−^^3^

### Special details

**Table:**

Experimental. CrysAlisPro 1.171.41.117a (Rigaku OD, 2021) Empirical absorption correction using spherical harmonics, implemented in SCALE3 ABSPACK scaling algorithm.
Geometry. All esds (except the esd in the dihedral angle between two l.s. planes) are estimated using the full covariance matrix. The cell esds are taken into account individually in the estimation of esds in distances, angles and torsion angles; correlations between esds in cell parameters are only used when they are defined by crystal symmetry. An approximate (isotropic) treatment of cell esds is used for estimating esds involving l.s. planes.

### Fractional atomic coordinates and isotropic or equivalent isotropic displacement parameters (Å^2^)

**Table:**

	*x*	*y*	*z*	*U*_iso_*/*U*_eq_	
N1	0.29981 (9)	0.24967 (9)	0.44133 (7)	0.0245 (2)	
H1	0.2870 (14)	0.2626 (14)	0.3869 (12)	0.029*	
C2	0.30095 (10)	0.32803 (10)	0.50096 (8)	0.0217 (2)	
O2	0.29321 (8)	0.42469 (7)	0.48817 (6)	0.0257 (2)	
C3	0.31293 (10)	0.27464 (10)	0.59181 (8)	0.0203 (2)	
C3A	0.33681 (10)	0.15908 (10)	0.56953 (8)	0.0207 (2)	
C4	0.36295 (10)	0.06941 (10)	0.61983 (8)	0.0241 (3)	
H4	0.3722	0.0747	0.6811	0.029*	
C5	0.37540 (12)	−0.02910 (10)	0.57855 (9)	0.0278 (3)	
H5	0.3942	−0.0911	0.6122	0.033*	
C6	0.36067 (12)	−0.03723 (11)	0.48910 (9)	0.0305 (3)	
H6	0.3694	−0.1049	0.4623	0.037*	
C7	0.33332 (12)	0.05214 (11)	0.43778 (9)	0.0288 (3)	
H7	0.3224	0.0465	0.3765	0.035*	
C7A	0.32274 (10)	0.14916 (10)	0.47930 (8)	0.0231 (3)	
N8	0.09577 (10)	0.13898 (10)	0.67133 (7)	0.0275 (2)	
H8	0.0674 (15)	0.0887 (15)	0.7013 (12)	0.033*	
C9	0.18227 (10)	0.20090 (10)	0.70137 (8)	0.0232 (3)	
O9	0.23955 (8)	0.18888 (8)	0.76917 (6)	0.0284 (2)	
C10	0.19781 (10)	0.29116 (10)	0.63396 (8)	0.0227 (3)	
C10A	0.09505 (11)	0.27542 (11)	0.57198 (8)	0.0257 (3)	
C11	0.04983 (12)	0.33584 (13)	0.50258 (10)	0.0346 (3)	
H11	0.0839	0.4011	0.4872	0.041*	
C12	−0.04637 (13)	0.29896 (16)	0.45597 (11)	0.0437 (4)	
H12	−0.0784	0.3396	0.4084	0.052*	
C13	−0.09603 (13)	0.20330 (16)	0.47827 (11)	0.0440 (4)	
H13	−0.1604	0.1785	0.4446	0.053*	
C14	−0.05321 (12)	0.14320 (14)	0.54893 (10)	0.0368 (3)	
H14	−0.0876	0.0782	0.5647	0.044*	
C14A	0.04124 (11)	0.18180 (11)	0.59521 (9)	0.0276 (3)	
C15	0.21617 (11)	0.39881 (10)	0.67678 (8)	0.0256 (3)	
C16	0.32451 (11)	0.43111 (10)	0.68444 (8)	0.0233 (3)	
N16	0.36972 (11)	0.51996 (9)	0.72048 (7)	0.0281 (2)	
H16A	0.4391 (16)	0.5352 (15)	0.7097 (12)	0.034*	
H16B	0.3234 (15)	0.5750 (15)	0.7399 (11)	0.034*	
C17	0.39908 (10)	0.34282 (10)	0.64964 (8)	0.0217 (2)	
C18	0.12832 (12)	0.45803 (11)	0.70963 (10)	0.0315 (3)	
N18	0.05723 (12)	0.50576 (12)	0.73688 (10)	0.0455 (3)	
C19	0.49892 (10)	0.38414 (10)	0.60184 (8)	0.0233 (3)	
O19	0.52472 (7)	0.31043 (7)	0.54332 (6)	0.0246 (2)	
N19	0.54541 (10)	0.47131 (10)	0.62336 (8)	0.0305 (3)	
H19	0.6035 (16)	0.4899 (15)	0.5939 (12)	0.037*	
C20	0.61787 (12)	0.33447 (11)	0.49283 (9)	0.0285 (3)	
H20A	0.6255	0.2776	0.4501	0.043*	
H20B	0.6865	0.3390	0.5312	0.043*	
H20C	0.6047	0.4031	0.4630	0.043*	
C21	0.45228 (11)	0.28321 (10)	0.72483 (8)	0.0239 (3)	
N21	0.50020 (10)	0.24235 (10)	0.78254 (8)	0.0322 (3)	

### Atomic displacement parameters (Å^2^)

**Table:**

	*U*^11^	*U*^22^	*U*^33^	*U*^12^	*U*^13^	*U*^23^
N1	0.0290 (5)	0.0262 (5)	0.0187 (5)	−0.0010 (4)	0.0053 (4)	0.0020 (4)
C2	0.0198 (5)	0.0246 (6)	0.0215 (6)	−0.0013 (4)	0.0063 (4)	0.0026 (4)
O2	0.0279 (5)	0.0229 (4)	0.0272 (5)	−0.0006 (3)	0.0074 (4)	0.0046 (3)
C3	0.0211 (5)	0.0201 (6)	0.0203 (5)	−0.0008 (4)	0.0064 (4)	0.0014 (4)
C3A	0.0200 (5)	0.0207 (6)	0.0222 (6)	−0.0017 (4)	0.0066 (4)	−0.0012 (4)
C4	0.0253 (6)	0.0236 (6)	0.0241 (6)	−0.0009 (5)	0.0058 (5)	0.0004 (5)
C5	0.0324 (7)	0.0212 (6)	0.0305 (7)	0.0006 (5)	0.0066 (5)	0.0013 (5)
C6	0.0361 (7)	0.0235 (6)	0.0329 (7)	−0.0010 (5)	0.0094 (6)	−0.0066 (5)
C7	0.0344 (7)	0.0293 (7)	0.0236 (6)	−0.0031 (5)	0.0074 (5)	−0.0057 (5)
C7A	0.0235 (6)	0.0244 (6)	0.0222 (6)	−0.0018 (5)	0.0059 (4)	−0.0001 (5)
N8	0.0279 (6)	0.0293 (6)	0.0263 (5)	−0.0066 (4)	0.0076 (4)	0.0047 (4)
C9	0.0247 (6)	0.0240 (6)	0.0223 (6)	0.0003 (5)	0.0098 (5)	−0.0009 (4)
O9	0.0339 (5)	0.0312 (5)	0.0206 (4)	−0.0012 (4)	0.0051 (4)	0.0002 (4)
C10	0.0227 (6)	0.0229 (6)	0.0237 (6)	0.0007 (4)	0.0083 (5)	0.0016 (5)
C10A	0.0222 (6)	0.0287 (6)	0.0271 (6)	0.0010 (5)	0.0074 (5)	0.0019 (5)
C11	0.0258 (6)	0.0418 (8)	0.0366 (7)	0.0021 (6)	0.0058 (6)	0.0119 (6)
C12	0.0295 (7)	0.0611 (11)	0.0398 (8)	0.0025 (7)	−0.0008 (6)	0.0153 (7)
C13	0.0255 (7)	0.0643 (11)	0.0414 (8)	−0.0069 (7)	−0.0033 (6)	0.0034 (8)
C14	0.0280 (7)	0.0443 (8)	0.0386 (8)	−0.0090 (6)	0.0053 (6)	0.0016 (6)
C14A	0.0238 (6)	0.0324 (7)	0.0273 (6)	−0.0019 (5)	0.0076 (5)	0.0018 (5)
C15	0.0292 (6)	0.0216 (6)	0.0273 (6)	0.0009 (5)	0.0113 (5)	−0.0003 (5)
C16	0.0296 (6)	0.0208 (6)	0.0210 (5)	0.0010 (5)	0.0100 (5)	0.0017 (4)
N16	0.0337 (6)	0.0211 (5)	0.0308 (6)	−0.0033 (4)	0.0120 (5)	−0.0041 (4)
C17	0.0243 (6)	0.0206 (6)	0.0208 (5)	−0.0004 (4)	0.0064 (4)	−0.0004 (4)
C18	0.0325 (7)	0.0273 (7)	0.0361 (7)	0.0007 (5)	0.0121 (6)	−0.0021 (5)
N18	0.0412 (7)	0.0417 (8)	0.0561 (8)	0.0086 (6)	0.0195 (6)	−0.0100 (6)
C19	0.0238 (6)	0.0243 (6)	0.0226 (6)	0.0004 (5)	0.0068 (4)	0.0003 (5)
O19	0.0257 (4)	0.0238 (4)	0.0256 (4)	−0.0020 (3)	0.0107 (3)	−0.0018 (3)
N19	0.0286 (6)	0.0289 (6)	0.0356 (6)	−0.0073 (5)	0.0127 (5)	−0.0051 (5)
C20	0.0298 (6)	0.0271 (6)	0.0306 (6)	−0.0017 (5)	0.0154 (5)	−0.0011 (5)
C21	0.0253 (6)	0.0227 (6)	0.0244 (6)	−0.0022 (5)	0.0059 (5)	−0.0029 (5)
N21	0.0343 (6)	0.0323 (6)	0.0296 (6)	0.0016 (5)	0.0006 (5)	−0.0005 (5)

### Geometric parameters (Å, º)

**Table:**

N1—C2	1.3442 (17)	C10A—C14A	1.3953 (19)
N1—C7A	1.4028 (17)	C11—C12	1.392 (2)
N1—H1	0.860 (18)	C11—H11	0.9500
C2—O2	1.2240 (16)	C12—C13	1.389 (3)
C2—C3	1.5534 (16)	C12—H12	0.9500
C3—C3A	1.5147 (16)	C13—C14	1.392 (2)
C3—C17	1.5655 (17)	C13—H13	0.9500
C3—C10	1.5878 (16)	C14—C14A	1.380 (2)
C3A—C4	1.3849 (17)	C14—H14	0.9500
C3A—C7A	1.4003 (17)	C15—C16	1.3582 (19)
C4—C5	1.3984 (18)	C15—C18	1.4148 (18)
C4—H4	0.9500	C16—N16	1.3368 (17)
C5—C6	1.3868 (19)	C16—C17	1.5429 (17)
C5—H5	0.9500	N16—H16A	0.88 (2)
C6—C7	1.393 (2)	N16—H16B	0.947 (19)
C6—H6	0.9500	C17—C21	1.4835 (17)
C7—C7A	1.3809 (18)	C17—C19	1.5461 (16)
C7—H7	0.9500	C18—N18	1.148 (2)
N8—C9	1.3477 (17)	C19—N19	1.2553 (18)
N8—C14A	1.4083 (18)	C19—O19	1.3441 (15)
N8—H8	0.87 (2)	O19—C20	1.4457 (15)
C9—O9	1.2188 (16)	N19—H19	0.89 (2)
C9—C10	1.5566 (17)	C20—H20A	0.9800
C10—C15	1.5060 (18)	C20—H20B	0.9800
C10—C10A	1.5158 (18)	C20—H20C	0.9800
C10A—C11	1.3882 (19)	C21—N21	1.1443 (18)
			
C2—N1—C7A	111.74 (11)	C14A—C10A—C10	108.39 (11)
C2—N1—H1	121.9 (12)	C10A—C11—C12	118.74 (14)
C7A—N1—H1	126.4 (12)	C10A—C11—H11	120.6
O2—C2—N1	127.46 (12)	C12—C11—H11	120.6
O2—C2—C3	124.71 (11)	C13—C12—C11	120.65 (15)
N1—C2—C3	107.83 (10)	C13—C12—H12	119.7
C3A—C3—C2	101.96 (9)	C11—C12—H12	119.7
C3A—C3—C17	121.25 (10)	C12—C13—C14	121.24 (15)
C2—C3—C17	107.21 (9)	C12—C13—H13	119.4
C3A—C3—C10	113.81 (10)	C14—C13—H13	119.4
C2—C3—C10	107.16 (9)	C14A—C14—C13	117.31 (15)
C17—C3—C10	104.59 (9)	C14A—C14—H14	121.3
C4—C3A—C7A	119.64 (11)	C13—C14—H14	121.3
C4—C3A—C3	132.76 (11)	C14—C14A—C10A	122.43 (13)
C7A—C3A—C3	107.53 (10)	C14—C14A—N8	127.74 (13)
C3A—C4—C5	118.67 (12)	C10A—C14A—N8	109.81 (12)
C3A—C4—H4	120.7	C16—C15—C18	123.36 (12)
C5—C4—H4	120.7	C16—C15—C10	114.15 (11)
C6—C5—C4	120.75 (12)	C18—C15—C10	122.43 (12)
C6—C5—H5	119.6	N16—C16—C15	129.59 (12)
C4—C5—H5	119.6	N16—C16—C17	120.64 (12)
C5—C6—C7	121.16 (12)	C15—C16—C17	109.63 (11)
C5—C6—H6	119.4	C16—N16—H16A	117.3 (12)
C7—C6—H6	119.4	C16—N16—H16B	120.3 (11)
C7A—C7—C6	117.48 (12)	H16A—N16—H16B	119.3 (16)
C7A—C7—H7	121.3	C21—C17—C16	108.02 (10)
C6—C7—H7	121.3	C21—C17—C19	103.92 (10)
C7—C7A—C3A	122.28 (12)	C16—C17—C19	114.99 (10)
C7—C7A—N1	127.58 (12)	C21—C17—C3	113.69 (10)
C3A—C7A—N1	110.12 (11)	C16—C17—C3	102.24 (10)
C9—N8—C14A	111.54 (11)	C19—C17—C3	114.17 (10)
C9—N8—H8	123.7 (12)	N18—C18—C15	179.49 (18)
C14A—N8—H8	123.1 (12)	N19—C19—O19	130.56 (12)
O9—C9—N8	126.50 (12)	N19—C19—C17	120.46 (11)
O9—C9—C10	125.30 (12)	O19—C19—C17	108.87 (10)
N8—C9—C10	108.20 (11)	C19—O19—C20	116.87 (10)
C15—C10—C10A	118.58 (11)	C19—N19—H19	116.0 (12)
C15—C10—C9	111.74 (10)	O19—C20—H20A	109.5
C10A—C10—C9	101.35 (10)	O19—C20—H20B	109.5
C15—C10—C3	101.22 (10)	H20A—C20—H20B	109.5
C10A—C10—C3	114.49 (10)	O19—C20—H20C	109.5
C9—C10—C3	109.63 (10)	H20A—C20—H20C	109.5
C11—C10A—C14A	119.54 (13)	H20B—C20—H20C	109.5
C11—C10A—C10	132.06 (13)	N21—C21—C17	174.75 (14)
			
C7A—N1—C2—O2	173.52 (12)	C9—C10—C10A—C14A	−7.41 (13)
C7A—N1—C2—C3	−7.02 (14)	C3—C10—C10A—C14A	110.49 (12)
O2—C2—C3—C3A	−171.59 (12)	C14A—C10A—C11—C12	−2.3 (2)
N1—C2—C3—C3A	8.93 (12)	C10—C10A—C11—C12	178.83 (14)
O2—C2—C3—C17	−43.22 (15)	C10A—C11—C12—C13	−0.2 (3)
N1—C2—C3—C17	137.30 (10)	C11—C12—C13—C14	1.8 (3)
O2—C2—C3—C10	68.59 (15)	C12—C13—C14—C14A	−0.8 (3)
N1—C2—C3—C10	−110.89 (11)	C13—C14—C14A—C10A	−1.8 (2)
C2—C3—C3A—C4	175.38 (13)	C13—C14—C14A—N8	176.05 (15)
C17—C3—C3A—C4	56.55 (18)	C11—C10A—C14A—C14	3.4 (2)
C10—C3—C3A—C4	−69.58 (17)	C10—C10A—C14A—C14	−177.49 (13)
C2—C3—C3A—C7A	−7.73 (12)	C11—C10A—C14A—N8	−174.82 (12)
C17—C3—C3A—C7A	−126.56 (11)	C10—C10A—C14A—N8	4.31 (15)
C10—C3—C3A—C7A	107.30 (11)	C9—N8—C14A—C14	−176.69 (14)
C7A—C3A—C4—C5	0.46 (18)	C9—N8—C14A—C10A	1.39 (16)
C3—C3A—C4—C5	177.04 (12)	C10A—C10—C15—C16	−141.23 (12)
C3A—C4—C5—C6	−0.7 (2)	C9—C10—C15—C16	101.48 (13)
C4—C5—C6—C7	0.1 (2)	C3—C10—C15—C16	−15.12 (14)
C5—C6—C7—C7A	0.7 (2)	C10A—C10—C15—C18	41.63 (17)
C6—C7—C7A—C3A	−1.0 (2)	C9—C10—C15—C18	−75.66 (16)
C6—C7—C7A—N1	177.60 (12)	C3—C10—C15—C18	167.73 (12)
C4—C3A—C7A—C7	0.41 (19)	C18—C15—C16—N16	−1.2 (2)
C3—C3A—C7A—C7	−176.96 (12)	C10—C15—C16—N16	−178.31 (12)
C4—C3A—C7A—N1	−178.41 (11)	C18—C15—C16—C17	174.49 (12)
C3—C3A—C7A—N1	4.22 (13)	C10—C15—C16—C17	−2.62 (15)
C2—N1—C7A—C7	−176.84 (13)	N16—C16—C17—C21	75.41 (14)
C2—N1—C7A—C3A	1.90 (15)	C15—C16—C17—C21	−100.73 (12)
C14A—N8—C9—O9	174.09 (12)	N16—C16—C17—C19	−40.11 (16)
C14A—N8—C9—C10	−6.31 (14)	C15—C16—C17—C19	143.75 (11)
O9—C9—C10—C15	−44.88 (17)	N16—C16—C17—C3	−164.39 (11)
N8—C9—C10—C15	135.52 (11)	C15—C16—C17—C3	19.47 (13)
O9—C9—C10—C10A	−172.13 (12)	C3A—C3—C17—C21	−41.76 (15)
N8—C9—C10—C10A	8.26 (13)	C2—C3—C17—C21	−157.97 (10)
O9—C9—C10—C3	66.51 (15)	C10—C3—C17—C21	88.47 (12)
N8—C9—C10—C3	−113.10 (11)	C3A—C3—C17—C16	−157.92 (10)
C3A—C3—C10—C15	160.56 (10)	C2—C3—C17—C16	85.87 (11)
C2—C3—C10—C15	−87.52 (11)	C10—C3—C17—C16	−27.69 (11)
C17—C3—C10—C15	26.07 (12)	C3A—C3—C17—C19	77.25 (14)
C3A—C3—C10—C10A	−70.67 (13)	C2—C3—C17—C19	−38.96 (13)
C2—C3—C10—C10A	41.26 (14)	C10—C3—C17—C19	−152.52 (10)
C17—C3—C10—C10A	154.85 (10)	C21—C17—C19—N19	−86.46 (15)
C3A—C3—C10—C9	42.42 (14)	C16—C17—C19—N19	31.40 (17)
C2—C3—C10—C9	154.34 (10)	C3—C17—C19—N19	149.14 (12)
C17—C3—C10—C9	−92.07 (11)	C21—C17—C19—O19	90.07 (11)
C15—C10—C10A—C11	48.9 (2)	C16—C17—C19—O19	−152.07 (11)
C9—C10—C10A—C11	171.58 (14)	C3—C17—C19—O19	−34.33 (14)
C3—C10—C10A—C11	−70.52 (18)	N19—C19—O19—C20	−3.0 (2)
C15—C10—C10A—C14A	−130.06 (12)	C17—C19—O19—C20	−179.10 (10)

### Hydrogen-bond geometry (Å, º)

**Table:**

*D*—H···*A*	*D*—H	H···*A*	*D*···*A*	*D*—H···*A*
N1—H1···O9^i^	0.860 (19)	1.963 (18)	2.8145 (14)	170.0 (16)
N8—H8···N18^ii^	0.866 (19)	2.110 (18)	2.9270 (19)	157.1 (17)
N16—H16*A*···N19	0.883 (19)	2.079 (19)	2.7531 (17)	132.5 (16)
N16—H16*A*···N21^iii^	0.883 (19)	2.684 (19)	3.1854 (17)	117.1 (14)
N19—H19···O2^iv^	0.894 (19)	2.130 (19)	2.9912 (15)	161.6 (17)
C11—H11···O2	0.95	2.53	3.1499 (18)	123
C20—H20*C*···O2^iv^	0.98	2.56	3.1938 (16)	123

Symmetry codes: (i) *x*, −*y*+1/2, *z*−1/2; (ii) −*x*, *y*−1/2, −*z*+3/2; (iii) −*x*+1, *y*+1/2, −*z*+3/2; (iv) −*x*+1, −*y*+1, −*z*+1.
